# Synthesis and characterization of zinc oxide thin films for optoelectronic applications

**DOI:** 10.1016/j.heliyon.2017.e00285

**Published:** 2017-04-04

**Authors:** E. Muchuweni, T.S. Sathiaraj, H. Nyakotyo

**Affiliations:** Department of Physics and Astronomy, Botswana International University of Science and Technology (BIUST), P. Bag 16, Palapye, Botswana

**Keywords:** Materials science, Nanotechnology, Condensed matter physics, Engineering

## Abstract

Micro-ring structured zinc oxide (ZnO) thin films were prepared on glass substrates by spray pyrolysis and their structural, morphological, optical and electrical properties were investigated. X-ray Diffraction (XRD) analysis revealed the films’ hexagonal wurtzite phase with a preferred (002) grain orientation. The mean crystallite size calculated on the basis of the Debye-Scherrer model was 24 nm and a small dislocation density of $1.7\times 10^{-3}\;\;nm^{-2}$ was obtained, indicating the existence of few lattice defects and good crystallinity. Scanning Electron Microscopy (SEM) micrographs revealed the film’s granular nature composed of rod-shaped and spherical nanoparticles which agglomerated to form micro-ring like film clusters on the film surface. The average transmittance in the visible region, optical band gap and Urbach energy were approximately 75–80%, 3.28 eV and 57 meV, respectively. The refractive index and extinction coefficient were determined using Swanepoel’s envelope method. Raman spectroscopy revealed the presence of small amounts of residual tensile stress and low density of defects in the ZnO thin films. This was consistent with XRD analysis. A low sheet resistivity $\left(6.03\times 10^{1}\;\;\Omega cm\right)$ and high figure of merit $\left(4.35\times 10^{-6}\;\;\Omega^{-1}\right)$ were obtained for our films indicating their suitability in optoelectronic applications.

## 1 Introduction

Transparent conducting oxide (TCO) thin films have attracted significant research attention in the recent years due to their wide use in optoelectronic devices such as touch screens, liquid crystal displays, solar cells and light emitting diodes [1, 2, 3]. TCOs should exhibit both high electrical conductivity and high optical transparency in the visible region. Indium tin oxide (ITO) is the most commonly used TCO due to its high transparency to visible light and high electrical conductivity [4, 5]. However, there has been a major concern to find suitable alternatives for ITO due to the high cost, scarcity and toxicity of indium, which is its principal constituent element [5]. Furthermore, the optical and electrical properties of ITO are degraded when exposed to a hydrogen plasma environment [3].

In this regard, transparent conducting zinc oxide (ZnO) is extremely an attractive replacement material for ITO due to its good optical and electrical properties coupled with the low cost, non-toxicity and abundance in nature of Zn [6]. ZnO is an n-type semiconductor material with a direct wide band gap energy (3.37 eV) and large exciton binding energy (60 meV) at room temperature [7]. Its other favourable properties include high electrochemical stability, high thermal stability and good stability in hydrogen plasma, compared to ITO [3, 8, 9, 10, 11]. Previous studies have also reported on the good electrical stability of ZnO thin films [12] and their use as an air stable anode for organic light emitting diodes (OLEDs) [13, 14].

An assortment of ZnO thin film nanostructures such as nanoparticles [15], nanorings [16], micro-rings [17, 18, 19, 20] and micro-rods [21] have been successfully synthesized using a variety of deposition techniques. The most explored deposition techniques include, radio frequency magnetron sputtering [22], electron beam evaporation [23], spin coating [24], dip coating [17, 25] and spray pyrolysis [15]. Out of these, spray pyrolysis is of particular interest due to its inexpensive cost, simplicity, no high vacuum requirement, safety, less waste production and capability of coating large areas [1].

Deliberate control of size and shape of ZnO nano/micro-structures is becoming an interesting field due to their inherent size and shape dependent applications [17] but to the best of our knowledge, only a few studies have reported on spray pyrolysis deposited ZnO microstructures. Therefore, preparation and characterization of such a material would contribute towards the fabrication of optoelectronic devices. In this study, we report the synthesis and characterization of ZnO thin films with micro-ring structures, deposited on glass substrates at 623 K by spray pyrolysis as possible candidates for replacing ITO films in optoelectronic applications.

## 2 Experimental

ZnO thin films with micro-ring structures were prepared on glass substrates (Corning NY 14831, USA) by spray pyrolysis from 0.1 M of zinc acetate dihydrate [Zn(CH_3_COO)_2_.2H_2_O] (Sigma-Aldrich, USA, 99.999% purity) dissolved in a mixture of methanol and deionized water. The ratio of methanol to deionized water was maintained at 2:1 and a few drops of acetic acid were added to the prepared solution to prevent the precipitation of zinc hydroxide. Prior to deposition, the substrates were ultrasonically cleaned with acetone, isopropanol and finally with deionized water for 15 min in each step and then dried using compressed air.

The prepared solution was pumped by means of an infusion syringe pump at a constant flow rate of 2 ml/min and sprayed directly onto glass substrates placed on a hot plate stove set at 623 K. A stream of compressed air was used to atomize the spray solution through the nozzle fixed at 15 cm directly above the substrate.

Film thickness was measured using a 2D surface profilometer (Alpha-step D-100, KLA-Tencor, USA) with a sub-angstrom resolution and 0.1% step height repeatability. Structural properties were examined by an X-ray diffractometer (XRD, D8 Advance, Bruker, Germany) using Cu Kα radiation (*λ* = 1.5418 Å), operated at 40 kV and 40 mA. The surface morphology was characterized by a Zeiss field-emission scanning electron microscope (FE-SEM) operating at 2.00 kV. Optical transmittance measurements were performed using a UV/Vis/NIR spectrophotometer (Lambda-750, Perkin-Elmer, America) in the 300–800 nm wavelength range. Electrical properties were evaluated from Current-Voltage (I–V) measurements using the four point probe equipment consisting of an EZ GP-4303 power supply, a Signatone probing Table and two Keithley 197 digital multimeters. Raman spectroscopy was performed using a Horiba–Jobin Yvon Raman Spectrometer (LabRAM HR Evolution, France) in the backscattering geometry with the 532 nm excitation line of a solid state laser at an incident power of 2 mW in the range 200–750 cm^−1^.

## 3 Results and discussion

### 3.1 Structural properties

**Fig. 1:**
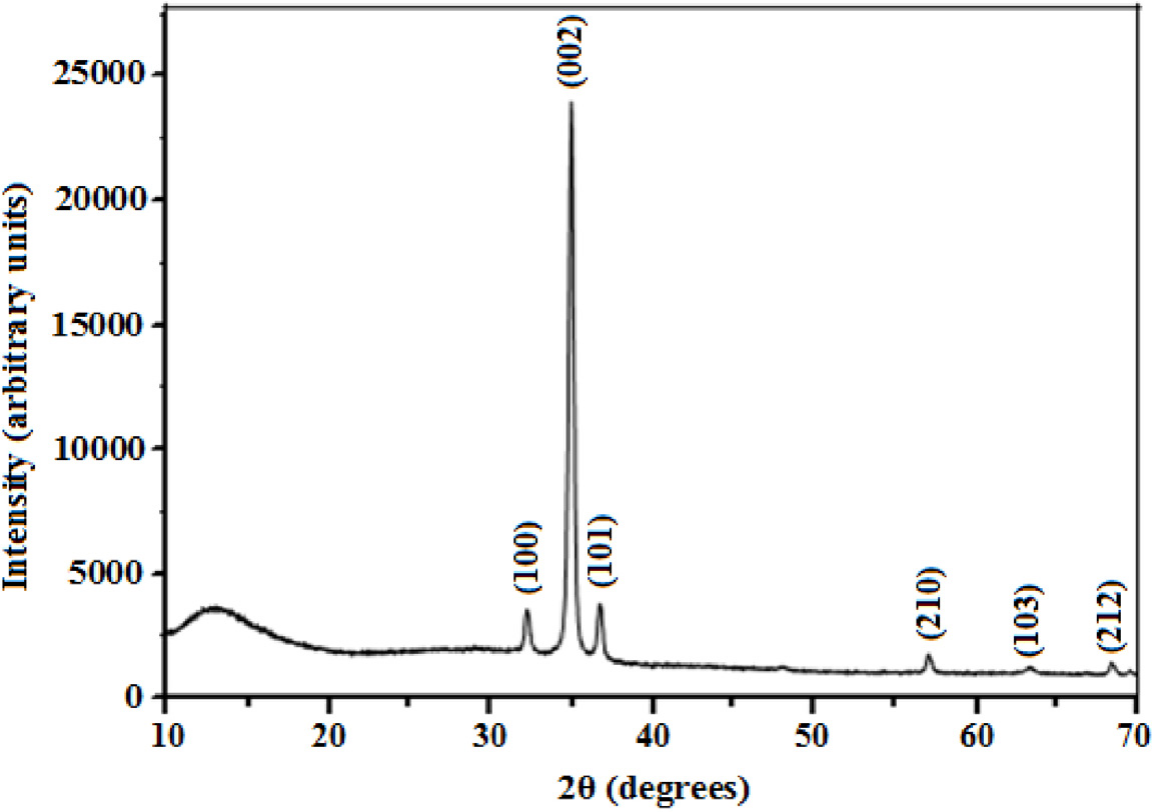
XRD pattern of the micro-ring structured ZnO thin film deposited on glass substrate at 623 K.

Fig. 1 shows the XRD pattern of micro-ring structured ZnO thin films of thickness 650 nm. All diffraction peaks were indexed to ZnO with a hexagonal wurtzite crystal structure (Crystallography Open Database, COD 10 11 258). The appearance of weak diffraction peaks corresponding to the (100), (101), (210), (103) and (212) planes of ZnO suggested the presence of some randomly oriented grains. However, there was a strong preferential growth orientation along the (002) plane indicating that the films grow perpendicular to the substrate. This was comparable with other ZnO microstructures such as micro-rings [20], microrods [26, 27] and microsausages [15] but in contrary with micro-rings [17, 19] which had a predominant (101) diffraction peak. Aslan et al. [28] reported that the (002) plane of ZnO is thermodynamically more favourable because it offers the least surface energy. The (002) peak position (35.018°) observed in this study was slightly higher than 34.467° for bulk ZnO (COD 10 11 258) and this was attributed to some residual stress in the film, likely originating from a mismatch in the thermal expansion coefficients of ZnO and the glass substrate. Liu et al. [29] attributed this deviation to surface effects which cause lattice deformations and reduction in the lattice parameter along the c-axis.

The mean crystallite size (*D*) was evaluated according to broadening of the highest intensity peak corresponding to the (002) diffraction plane using the Debye-Scherrer formula shown in Eq. (1) [30]:

(1) $$D=\frac{0.9\lambda }{\beta COS\theta }$$

where *λ*, β, and *θ* are the X-ray wavelength (1.5418 Å), full width at half maximum (FWHM) in radians (corrected for instrumental broadening) and Bragg’s diffraction angle, respectively. The FWHM and *D* were found to be 0.347° and 24 nm, respectively. The dislocation density (δ) was calculated from *D* using Eq. (2) [31]:

(2) $$\delta =\frac{1}{D^{2}}$$

and was found to be $1.7\times 10^{-3}nm^{-2}$. This lower value for δ implied that our films had very few lattice defects and good crystalline qualities. The lattice parameters *a* and *c* were calculated using Eq. (3) [32]:

(3) $$\frac{1}{d_{hkl}^{2}}=\frac{4}{3}\left(\frac{h^{2}+hk+k^{2}}{a^{2}}\right)+\frac{l^{2}}{c^{2}}$$

where $d_{hkl}$ is the interplanar spacing obtained from Bragg’s law, and *h, k* and *l* are the Miller indices denoting the plane. The lattice parameters were estimated to be *a* = 3.21 Å and *c* = 5.12 Å, which are slightly lower than those for bulk ZnO, *a* = 3.22 Å and *c* = 5.2 Å (COD 10 11 258), may be due to the effects of compressive strain on the film’s grains.

The strain (ε) and stress (σ) in the film, along the c-axis were found to be $-1.45\times 10^{-2}$ and 3.37  GPa, respectively, using Eqs. (4) and (5) [33, 34]:

(4) $$\varepsilon =\frac{c_{film}-c_{bulk}}{c_{bulk}}$$

(5) $$\sigma =-2.33\times 10^{11}\left(\frac{c_{film}-c_{bulk}}{c_{bulk}}\right)$$

where $c_{film}$ and *c_bulk_* (5.2 Å) are the lattice parameters of the film and unstrained ZnO, respectively. The obtained values indicated the presence of small amounts of compressive strain and tensile stress in the film and these may be the ones responsible for the slightly larger (002) peak position and relatively smaller lattice parameter c. The Zn-O bond length (*L*) was also calculated using Eqs. (6) and (7) [35]:

(6) $$L=\sqrt{\left(\frac{a^{2}}{3}+{\left(\frac{1}{2}-u\right)}^{2}c^{2}\right)}$$

(7) $$u=\frac{a^{2}}{3c^{2}}+\frac{1}{4}$$

where *u* is a wurtzite structure parameter. L was found to be 1.95 Å, which is slightly lower than 1.9767 Å for bulk ZnO [36] and this may be a result of compressive strain on the film.

### 3.2 Morphological properties

**Fig. 2:**
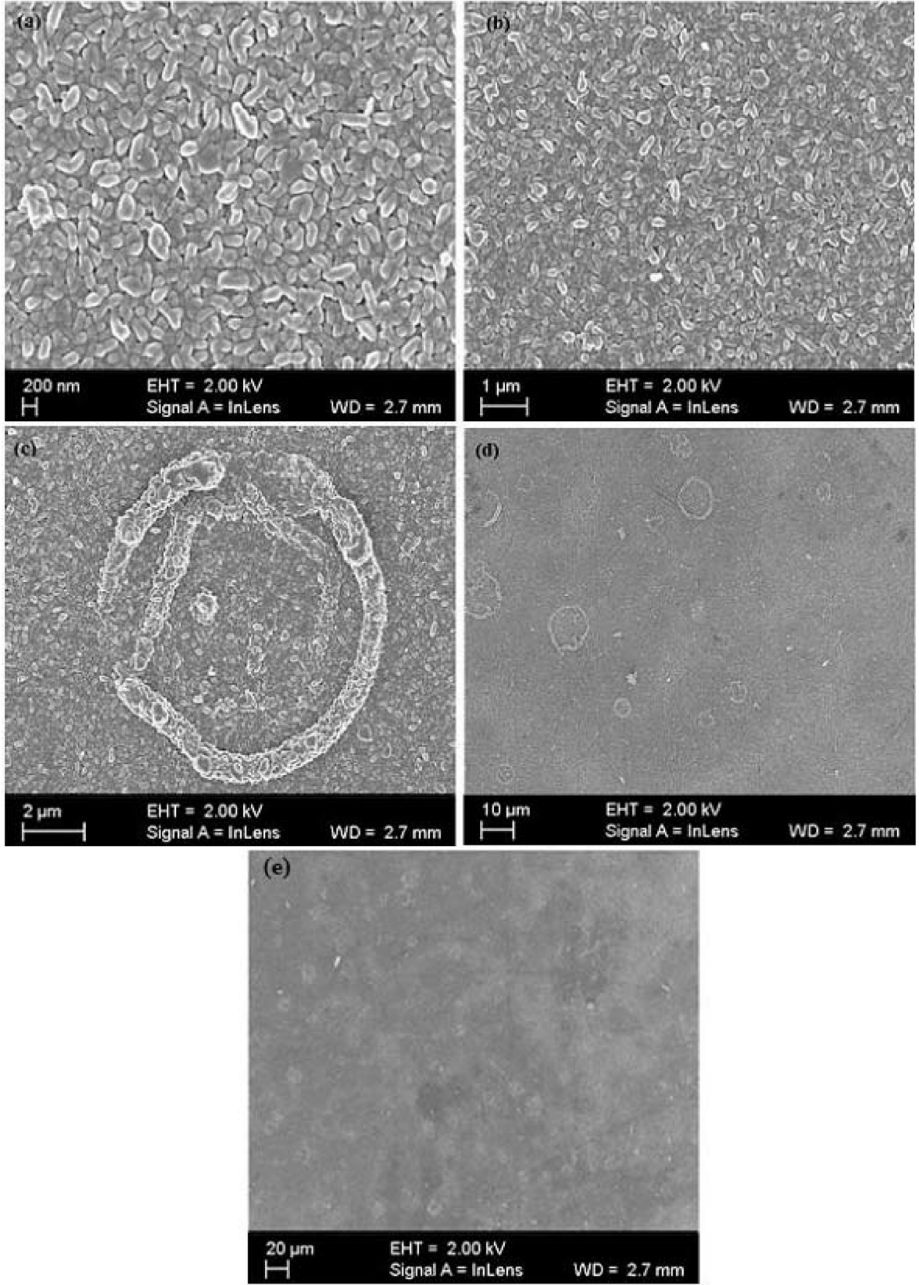
The SEM micrographs obtained at resolutions of (a) 200 nm, (b) 1 μm, (c) 2 μm, (d) 10 μm and (e) 20 μm, respectively.

Fig. 2 shows SEM micrographs that were taken at resolutions of (a) 200 nm, (b) 1 μm, (c) 2 μm, (d) 10 μm and (e) 20 μm, respectively. All samples had a uniform crack free structure. Fig. 2(a) and (b) revealed the film’s granular nature consisting of uniformly distributed rod-shaped and spherical nanoparticles of around 400 nm length and 100 nm diameter, respectively. Fig. 2(c) − (e) revealed that the microstructure was not completely flat due to nanoparticles which piled up to form micro-ring structures of diameter approximately, 5–10 μm. This was comparable with ZnO micro-rings by Hossain et al. [17] which had diameters of 5–13 μm.

### 3.3 Optical properties

**Fig. 3:**
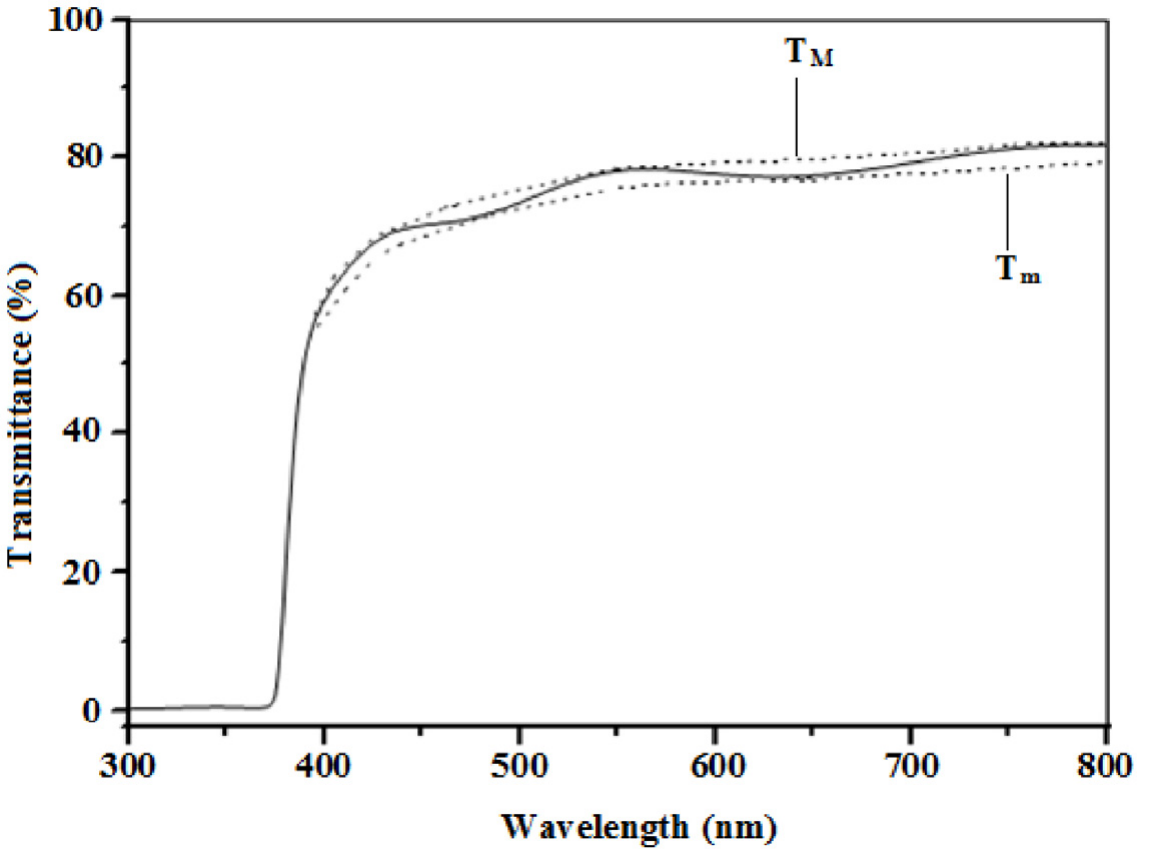
Optical transmission spectrum of the micro-ring structured ZnO thin film. The dotted curves for T_M_ and T_m_ are the envelopes generated in the high transmission region.

Fig. 3 shows the transmittance spectrum of the ZnO micro-ring structured thin films. Fluctuations and wave-like patterns appeared on the transmittance spectrum due to the interference of light reflected between the air-film and film-glass interfaces, indicating the film’s low surface roughness and good uniformity [37]. The film had an average transparency of about 75–80% in the visible region which may be associated with the film’s good structural homogeneity and crystallinity [7]. This was higher than 20% [26] and 25% [27] for undoped ZnO microrods and 60% [15] for undoped ZnO microsausages. A sharp absorption edge was observed at approximately 375 nm and this was in fair agreement with Karakaya et al. [38] and Winer et al. [10]. This strong absorption correlates with the band gap at which incident photons will be having enough energy to cause electronic excitations across the band gap [38].

**Fig. 4:**
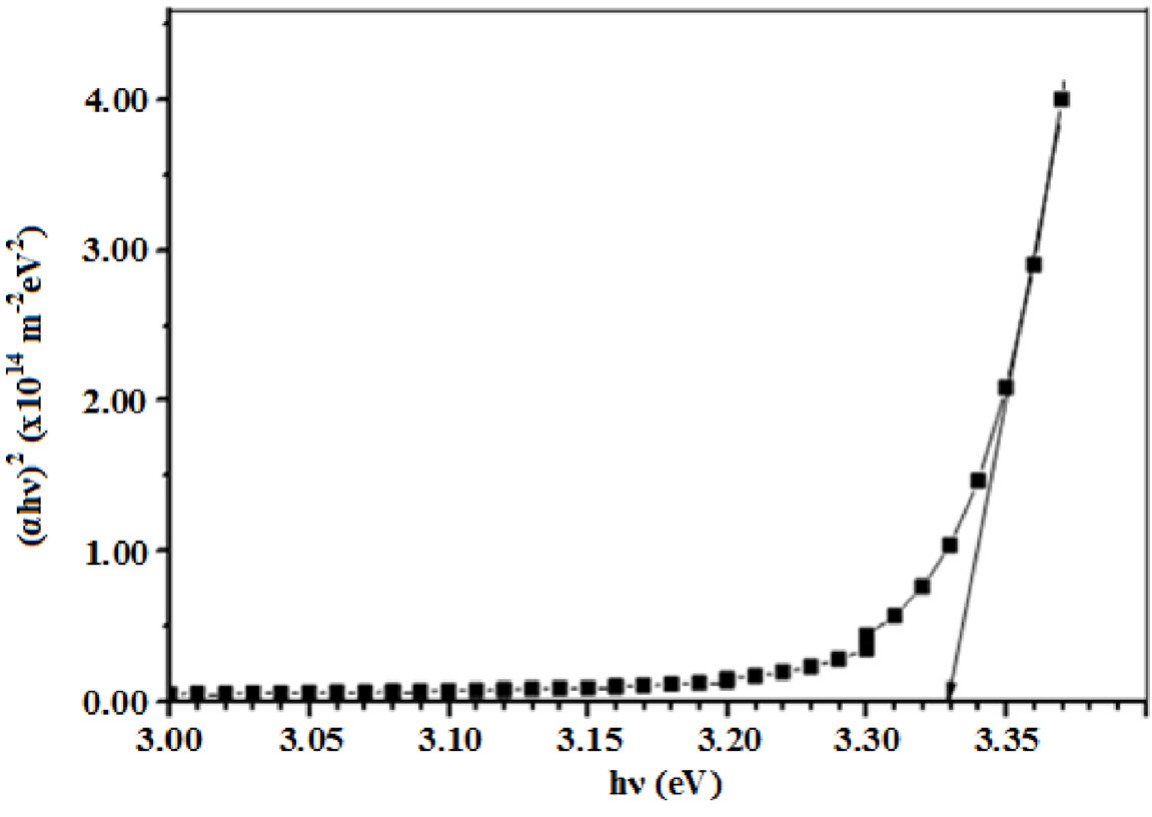
Variation of ${\left(\alpha hv\right)}^{2}$ versus hv for the micro-ring structured ZnO thin film.

The optical absorption coefficient (α) was calculated in the strong absorption region using the Beer-Lambert law shown in Eq. (8) [39]:

(8) $$\alpha =\frac{1}{t}ln\left[\frac{1}{T}\right]$$

where t and T are the film thickness and transmittance, respectively. The optical band gap (*E_g_*) was estimated by assuming a direct transition between the valence and conduction bands using the Tauc model in the high absorption region, given in Eq. (9) [40]:

(9) $${\left(\alpha hv\right)}^{2}=B\left(hv-E_{g}\right)$$

where *hν* is the energy of the incident photon and *B* is an energy-independent constant. *E_g_* was found to be 3.28 eV from the Tauc plot of ${\left(\alpha hv\right)}^{2}$ versus hv, shown in Fig. 4, by extrapolating the linear portion of the absorption edge to (αhv)=0. This was slightly less than 3.31 eV for bulk ZnO [41], due to grain boundaries and imperfections in the film [42]. However, this value was relatively greater than 3.22 eV for ZnO micro-rings [20], 3.15 eV [26] and 3.26 eV [24] for ZnO microrods and 3.18 eV [15] for ZnO microsausages.

**Fig. 5:**
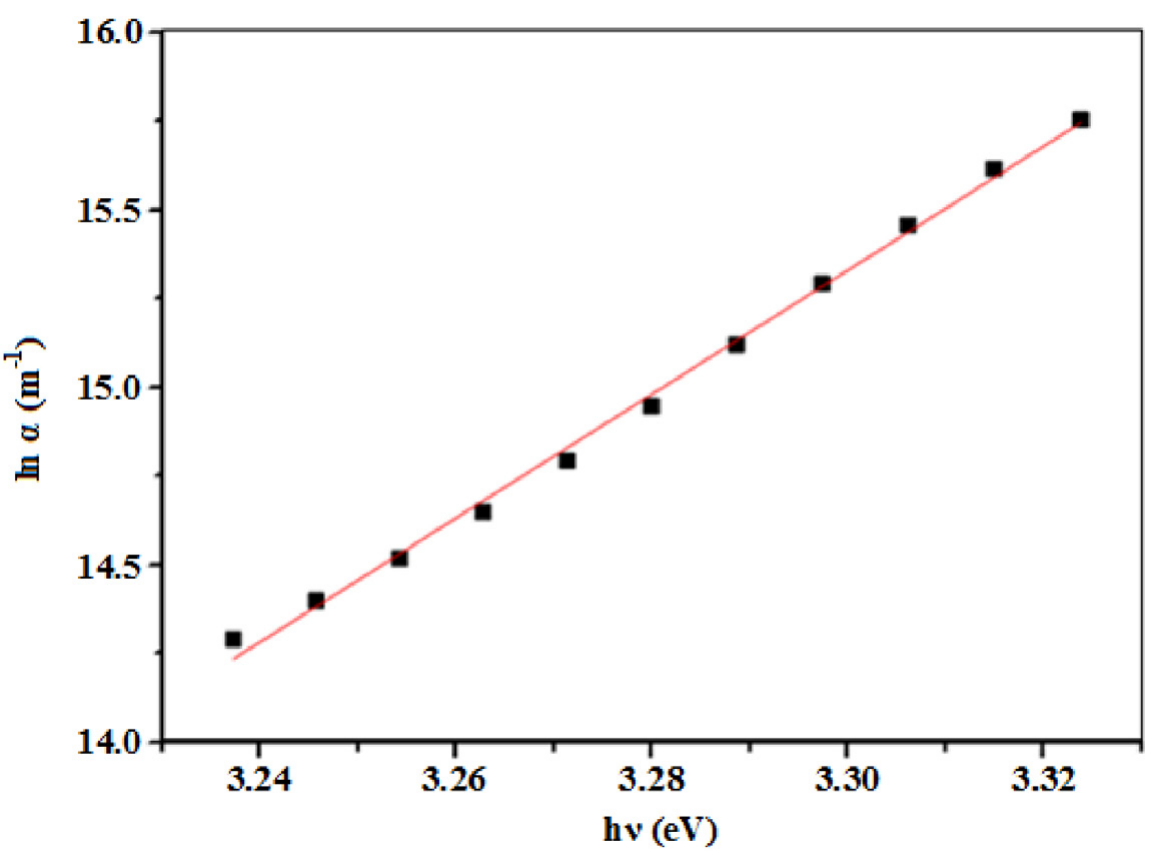
A plot of lnα versus hv of the ZnO micro-ring structured thin film.

Near the fundamental absorption edge, the absorption coefficient shows an exponential dependence on photon energy as shown in Eq. (10) [43]:

(10) $$\alpha =\alpha_{0}exp\left(\frac{hv}{E_{u}}\right)$$

where $\alpha_{0}$ is a constant and *E_u_* is the Urbach energy interpreted as the width of the exponential absorption edge. In the Urbach absorption tail model, the absorption edge band gap of a material arises from states created by that material’s defects and disorders [14]. Caglar et al. [44] reported that *E_u_* plays a significant role in investigating structural disorders in a thin film. Fig. 5 shows a linear fit of lnα versus hv on which *E_u_* was found to be 57 meV by calculating the reciprocal of the gradient.

The refractive index (*n)* and extinction coefficient (*k)* were calculated from the transmittance spectrum (Fig. 4) using Swanepoel’s envelope method. Sathiaraj [4], reported on the relative stability of the envelope method and its immunity to imprecision in measured data. The refractive index was calculated from Eqs. (11) and (12) [45]:

(11) $$n=\sqrt{N+\sqrt{\left(N^{2}-n_{s}^{2}\right)}}$$

(12) $$N=\frac{\left(n_{s}^{2}+1\right)}{2}+2n_{s}\frac{\left(T_{M}-T_{m}\right)}{T_{M}T_{m}}$$

where $n_{s}=1.52$ is the refractive index of the glass substrate, *T_M_* and *T_m_* are maximum and minimum transmittances measured at the same wavelength in the fitted envelopes on Fig. 4. The extinction coefficient was also calculated from Eqs. (13) and (14) [38, 39]:

(13) $$k=\frac{\alpha \lambda }{4\pi }$$

**Fig. 6:**
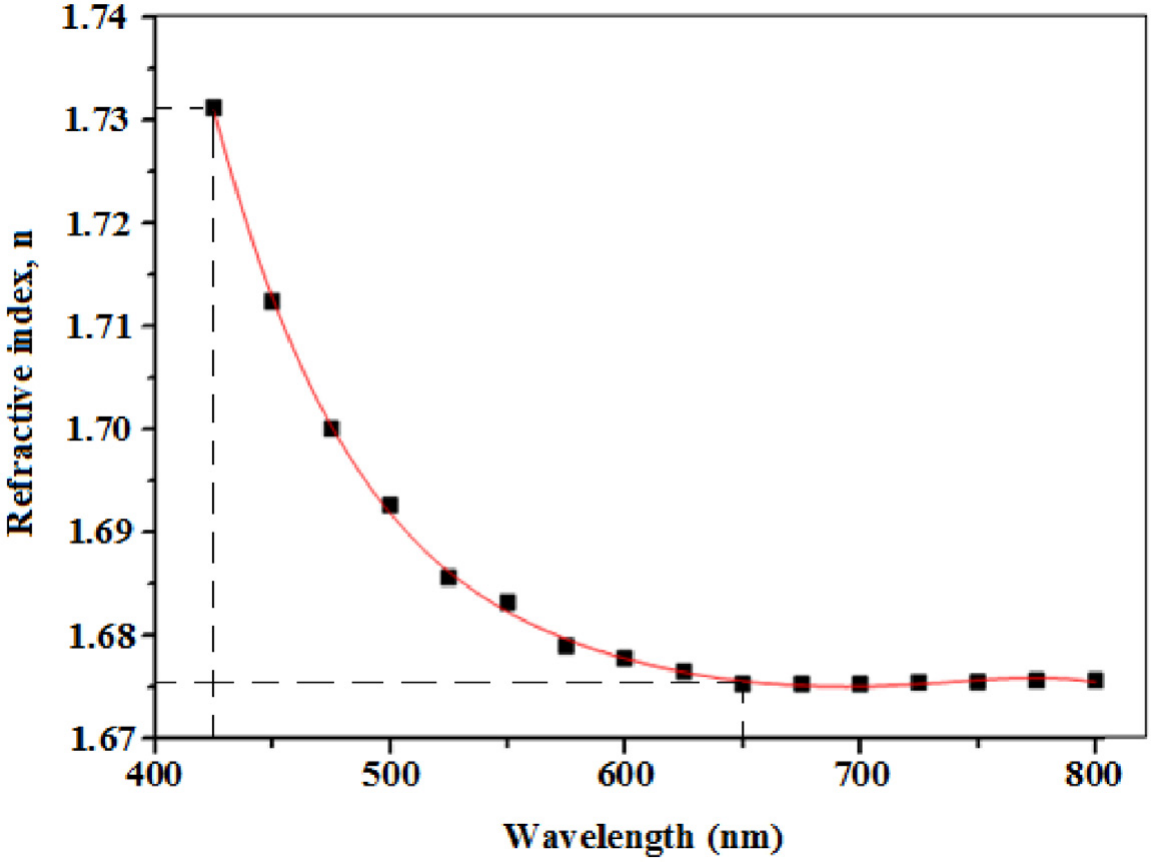
The dependence of refractive index with wavelength.

**Fig. 7:**
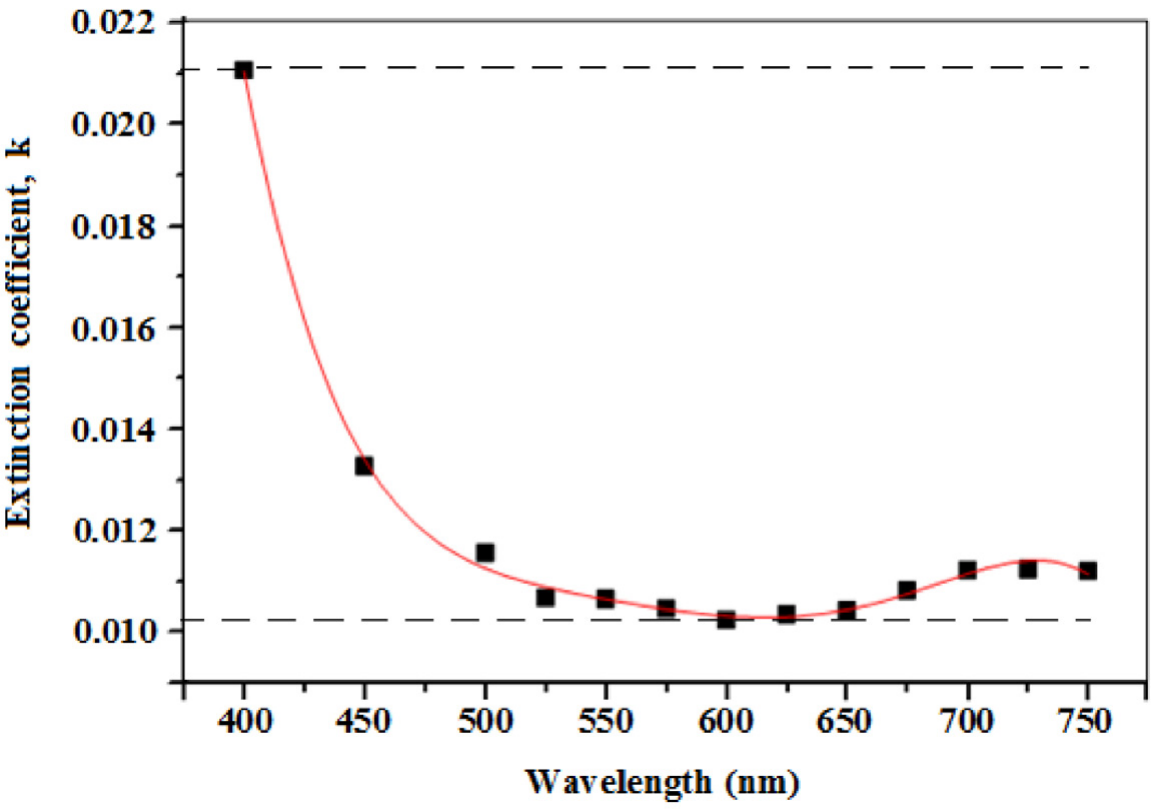
The dependence of extinction coefficient with wavelength.

(14) α=1tln(n−1)(n−ns)(n+1)(n−ns)[(TMTm)+1(TMTm)−1]

where α is the absorption coefficient and t is the film thickness. The spectral dependence of both *n* and *k* in the visible region was respectively plotted in Fig. 6 and Fig. 7.

The refractive index was observed to decrease exponentially from about 1.731 to 1.675 between 425 nm and 650 nm, after which it became nearly constant. Small *k* values between 0.010 and 0.021 were obtained over the entire visible region, indicating the high transparency nature of the prepared films. The spectral dependence of *n* and *k* with wavelength obtained in our study is in good agreement with Ashour et al. [46].

### 3.4 Electrical properties

**Fig. 8:**
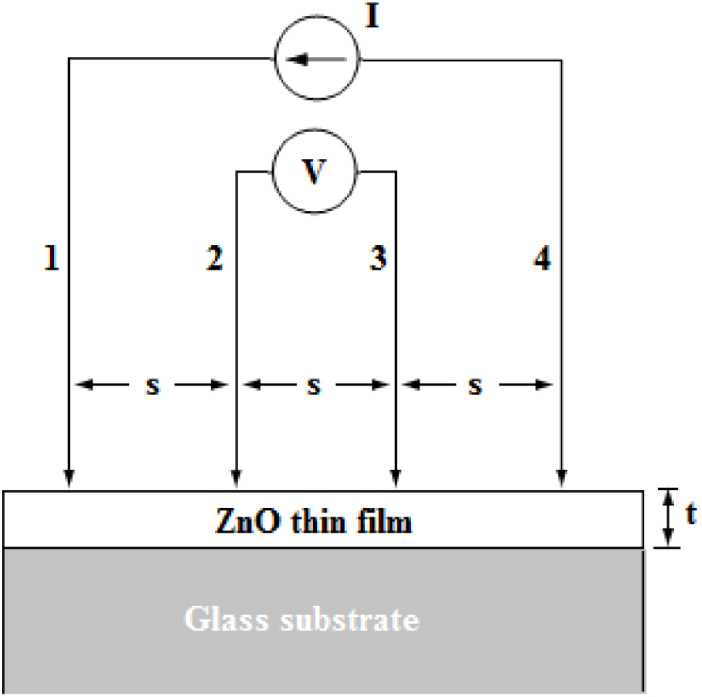
Schematic diagram of the Four-Point Probe used to determine the electrical resistivity of the ZnO thin film of thickness 650 nm, on a glass substrate of dimensions, 75 mm x 25 mm x 1 mm.

The schematic representation of the Four-Point Probe (shown in Fig. 8) illustrates that a fixed current (I) is injected into the ZnO thin film of thickness (t) through the two outer probes (1 and 4) and a voltage (V) is measured between the two inner probes (2 and 3).

For probes with a uniform spacing s (1.6 mm) >> t (650 nm), the electrical resistivity (ρ) is given by Eq. (15) [47]:

(15) $$\rho =4.532\left(\frac{V}{I}\right)t=R_{s}t$$

where V, I, R_s_ and t are the voltage, current, sheet resistance and film thickness, respectively. The electrical resistivity was found to be $6.03\times 10^{1}\;\;\Omega cm$, which is relatively lower than $5.39\times 10^{3}\;\;\Omega cm$ and $2.23\times 10^{3}\;\;\Omega cm$ reported by Muiva et al. [15] and Bacaksiz et al. [26], respectively, for undoped ZnO microstructured thin films. Generally, the electrical resistivity of thin films may be affected by isotropic background scattering (arising from phonons and point defects), external surface scattering and grain boundary scattering [48]. In this study, the electrical resistivity was likely influenced by carrier scattering and trapping at grain boundaries of the small sized particles rather than scattering due to crystal defects since a very small defect density value was obtained. Absence of doping may also have caused the observed electrical resistivity which is in the order of $10^{1}\;\;\Omega cm$. Dahoudi [11] reported that intergranular voids and pores present discontinuities between nanoparticles, thereby inhibiting the smooth mobility of charge carriers, resulting in a relatively high electrical resistivity. Therefore, in this study the appearance of very few pores and voids as revealed by SEM analysis, presented less discontinuities between nanoparticles, hence, giving rise to the obtained low electrical resistivity.

The suitability of our films for optoelectronic applications was quantified by the best combination of high electrical conductivity and low absorption of visible light, known as the figure of merit (ϕ), calculated using Eq. (16) [49]:

(16) $$\phi =\frac{1}{\alpha \rho }$$

where α is the absorption coefficient at 550 nm and *ρ* is the electrical resistivity. The figure of merit for our films was $\left(4.35\times 10^{-6}\;\;\Omega^{-1}\right)$. This value is relatively higher than $1.5\times 10^{-6}\;\;\Omega^{-1}$ obtained for spray deposited ZnO microstructures by Muiva et al. [15] which they reported as acceptable for optoelectronic applications, indicating that our films are also suitable for optoelectronic device fabrication.

### 3.5 Raman spectroscopy

**Fig. 9:**
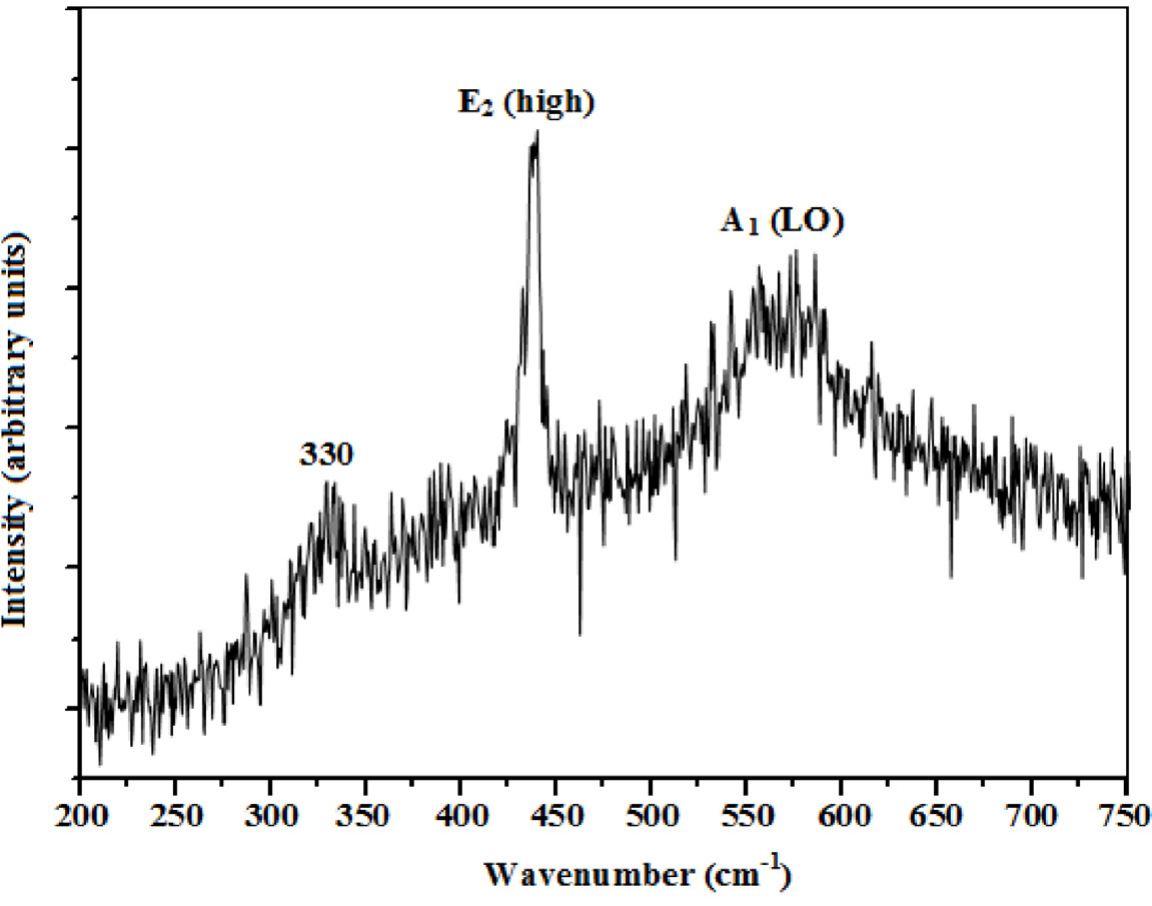
Raman spectrum of the ZnO thin film with micro-ring structures.

Fig. 9 shows the Raman spectrum of the ZnO thin films. The Raman peaks around 437 cm^−1^ and 575 cm^−1^ were assigned to ZnO *E_2_* (high) and A*_1_* longitudinal optical (LO) modes, respectively. Raman spectroscopy of the *E_2_* phonons plays a significant role in the study of residual stress within ZnO crystals because the stress induced in wurtzite structure crystals affects the *E_2_* phonon frequency, hence, allowing the extraction of information on stress from the *E_2_* mode [50, 51]. A decrease in the *E_2_* phonon frequency is attributed to tensile stress while its increase is attributed to compressive stress [50, 51]. In the present study, the *E_2_* vibration mode at 437 cm^−1^ is characteristic of the wurtzite phase and its value is slightly lower than 439 cm^−1^ for the stress free bulk ZnO [52, 53], suggesting that our ZnO thin films were under some slight tensile stress. The stress probably originated from a mismatch in the thermal expansion coefficient of the ZnO thin film $\left(4.75\times 10^{-6}\;\;{\text{K}}^{-1}\right)$ and the glass substrate $\left(2.60\times 10^{-6}\;\;{\text{K}}^{-1}\right)$ [54]. The relatively higher intensity and sharp peak of the *E_2_* (high) mode as compared to the other peaks also indicated that the ZnO thin films had a hexagonal wurtzite phase with good crystallinity. This was consistent with XRD analysis.

The A*_1_* (LO) mode at 575 cm^−1^ originates from defects such as oxygen vacancies and Zn interstitials [55] and its relatively low intensity peak indicates a relatively low density of defects in the ZnO thin films. This corroborates our XRD analysis. In addition, the position of the A*_1_* (LO) mode obtained in this study was comparable with that of bulk ZnO (574 cm^−1^) [52, 53]. The weak peak at 330 cm^−1^ was attributed to second order Raman processes [52, 53].

## 4 Conclusion

The structural, morphological, optical and electrical properties of spray pyrolysis deposited ZnO micro-ring structured thin films were characterized. The films had a hexagonal wurtzite crystal structure with a preferred orientation along the (002) direction. SEM micrographs showed nanoparticles which coalesced to form micro-rings on the surface of the film. High average transmittances, around 75–80% were observed in the visible region. The wavelength dependence of refractive index and extinction coefficient also confirmed the high transparency nature of the films. The optical band gap and Urbach energy were determined to be 3.28 eV and 57 meV, respectively. The ZnO *E_2_* (high) and A*_1_* (LO) Raman modes were observed at 436 cm^−1^ and 576 cm^−1^, respectively. A low electrical resistivity of $6.03\times 10^{1}\;\;\Omega cm$ and high figure of merit of $4.35\times 10^{-6}\;\;\Omega^{-1}$, indicated the suitability of the ZnO micro-ring structured thin films for optoelectronic applications.

## Declarations

### Author contribution statement

Edigar Muchuweni: Conceived and designed the experiments; Performed the experiments; Analyzed and interpreted the data; Contributed reagents, materials, analysis tools or data; Wrote the paper.

Thangiah S. Sathiaraj, Huggins Nyakotyo: Analyzed and interpreted the data; Contributed reagents, materials, analysis tools or data.

### Funding statement

This research did not receive any specific grant from funding agencies in the public, commercial, or not-for-profit sectors.

### Competing interest statement

The authors declare no conflict of interest.

### Additional information

No additional information is available for this paper.

## References

[1] Muchuweni E., Sathiaraj T.S., Nyakotyo H. (2016). Effect of gallium doping on the structural, optical and electrical properties of zinc oxide thin films prepared by spray pyrolysis. Ceram. Int..

[2] Muchuweni E., Sathiaraj T.S., Nyakotyo H. (2016). Low temperature synthesis of radio frequency magnetron sputtered gallium and aluminium co-doped zinc oxide thin films for transparent electrode fabrication. Appl. Surf. Sci..

[3] Lee J.−H., Ko K.−H., Park B.−O. (2003). Electrical and optical properties of ZnO transparent conducting films by the sol-gel method. J. Cryst. Growth.

[4] Sathiaraj T.S. (2008). Effect of annealing on the structural, optical and electrical properties of ITO films by RF sputtering under low vacuum level. Microelectron. J..

[5] Kumar Srivastava A., Kumar J. (2013). Effect of zinc addition and vacuum annealing time on the properties of spin-coated low-cost transparent conducting 1 at% Ga-ZnO thin films. Sci. Technol. Adv. Mater..

[6] Muchuweni E., Sathiaraj T.S., Nyakotyo H. (2016). Physical properties of gallium and aluminium co-doped zinc oxide thin films deposited at different radio frequency magnetron sputtering power. Ceram. Int..

[7] Shinde S.S., Shinde P.S., Oh Y.W., Haranath D., Bhosale C.H., Rajpure K.Y. (2012). Structural optoelectronic, luminescence and thermal properties of Ga-doped zinc oxide thin films. Appl. Surf. Sci..

[8] Sahal M., Hartiti B., Ridah A., Mollar M., Marí B. (2008). Structural, electrical and optical properties of ZnO thin films deposited by sol-gel method. Microelectron. J..

[9] Muiva C.M., Sathiaraj T.S., Maabong K. (2011). Effect of doping concentration on the properties of aluminium doped zinc oxide thin films prepared by spray pyrolysis for transparent electrode applications. Ceram. Int..

[10] Winer I., Shter G.E., Mann-Lahav M., Grader G.S. (2011). Effect of solvents and stabilizers on sol–gel deposition of Ga-doped zinc oxide TCO films. J. Mater. Res..

[11] Dahoudi N.A. (2014). Comparative study of highly dense aluminium- and gallium-doped zinc oxide transparent conducting sol–gel thin films. Bull. Mater. Sci..

[12] Lu H., Wang Y., Lin X. (2009). Structures, varistor properties and electrical stability of ZnO thin films. Mater. Lett..

[13] Rao T.P., Kumar M.C.S. (2012). Resistivity Stability of Ga Doped ZnO Thin Films with Heat Treatment in Air and Oxygen Atmospheres. J. Cryst. Process Technol..

[14] Rao T.P., Kumar M.C.S. (2010). Physical properties of Ga-doped ZnO thin films by spray pyrolysis. J. Alloy. Compd..

[15] Muiva C., Sathiaraj T.S., Maabong K. (2012). Chemical Spray Pyrolysis Path to Synthesis of ZnO Microsausages from Aggregation of Elongated Double Tipped Nanoparticles. Mater. Sci. Forum.

[16] Hughes W.L., Wang Z.L. (2005). Controlled synthesis and manipulation of ZnO nanorings and nanobows. Appl. Phys. Lett..

[17] Hossain M.F., Zhang Z.H., Takahashi T. (2010). Novel micro-ring structured ZnO photoelectrode for dye-sensitized solar cell. Nano-Micro Lett..

[18] Maciąg A., Sagan P., Kuźma M., Popovych V. (2017). Zinc oxide films prepared by spray pyrolysis. EPJ Web. Conf..

[19] Noh Y.−J., Na S.−I., Kim S.−S. (2013). Inverted polymer solar cells including ZnO electron transport layer fabricated by facile spray pyrolysis. Sol. Energ. Mat. Sol. Cells.

[20] Yakuphanoglu F. (2010). Electrical characterization and device characterization of ZnO microring shaped films by sol-gel method. J. Alloy. Compd..

[21] Alver U., Kilinç T., Bacaksiz E., Küçükömeroğlu T., Nezir S., Mutlu I.H., Aslan F. (2007). Synthesis and characterization of spray pyrolysis Zinc Oxide microrods. Thin Solid Films.

[22] Nunes P., Costa D., Fortunato E., Martins R. (2002). Performances Presented by Zinc Oxide Thin Films Deposited by R. F. Magnetron Sputtering. Vacuum.

[23] Choi W.S., Kim E.J., Seong S.G., Kim Y.S., Park C., Hahn S.H. (2009). Optical and structural properties of ZnO/TiO2/ZnO multi-layers prepared via electron beam evaporation. Vacuum.

[24] Caglar M., Ilican S., Caglar Y., Yakuphanoglu F. (2009). Electrical conductivity and optical properties of ZnO nanostructured thin film. Appl. Surf. Sci..

[25] Kaneva N.V., Dushkin C.D. (2011). Preparation of nanocrystalline thin films of ZnO by sol-gel dip coating. Bulg. Chem. Commun..

[26] Bacaksız E., Aksu S., Yılmaz S., Parlak M., Altunbaş M. (2010). Structural, optical and electrical properties of Al-doped ZnO microrods prepared by spray pyrolysis. Thin Solid Films.

[27] Yılmaz S., Bacaksız E., McGlynn E., Polat İ., Özcan Ş. (2012). Structural, optical and magnetic properties of Zn1-xMnxO micro-rod arrays synthesized by spray pyrolysis method. Thin Solid Films.

[28] Aslan M.H., Oral A.Y., Menşur E., Gül A., Başaran E. (2004). Preparation of c-axis-oriented zinc oxide thin films and the study of their microstructure and optical properties. Sol. Energy Mater. Sol. Cells.

[29] Liu Y.X., Liu Y.C., Shen D.Z., Zhong G.Z., Fan X.W., Kong X.G., Mu R., Henderson D.O. (2002). Preferred orientation of ZnO nanoparticles formed by post-thermal annealing implanted silica. Solid State Commun..

[30] Maniv S., Zangvil A. (1978). Controlled texture of reactively rf-sputtered ZnO thin films. J. Appl. Phys..

[31] Wang X.S., Wu Z.C., Webb J.F., Liu Z.G. (2003). Ferroelectric and dielectric properties of Li-doped ZnO thin films prepared by pulsed laser deposition. Appl. Phys. A.

[32] Warren B.E. (1990). X-ray diffraction.

[33] Şenadim Tüzemen E., Kavak H., Esen R. (2007). Influence of oxygen pressure of ZnO/glass substrate produced by pulsed filtered cathodic vacuum arc deposition. Phys. B: Condens. Matter.

[34] Wang Y.G., Lau S.P., Lee H.W., Yu S.F., Tay B.K., Zhang X.H., Tse K.Y., Hng H.H. (2003). Comprehensive study of ZnO films prepared by filtered cathodic vacuum arc at room temperature. J. Appl. Phys..

[35] Aksoy S., Caglar Y., Ilican S., Caglar M. (2010). Effect of Deposition Temperature on the Crystalline Structure and Surface Morphology of ZnO Films Deposited on p–Si, Adv. Control. Chem. Eng. Civ. Eng. Mech. Eng..

[36] Seetawan U., Jugsujinda S., Seetawan T., Ratchasin A., Euvananont C., Junin C., Thanachayanont C., Chainaronk P. (2011). Effect of calcinations temperature on crystallography and nanoparticles in ZnO disk. Mater. Sci. Appl..

[37] You Z.Z., Hua G.J. (2012). Electrical, optical and microstructural properties of transparent conducting GZO thin films deposited by magnetron sputtering. J. Alloy. Compd..

[38] Karakaya S., Ozbas O. (2015). Preparation and Characterization of Highly Conducting and Transparent Conducting ZnO Thin Films by Ultrasonic Spray Pyrolysis. Can. J. Basic Appl. Sci..

[39] Gümüş C., Ozkendir O.M., Kavak H., Ufuktepe Y. (2006). Structural and optical properties of zinc oxide thin films prepared by spray pyrolysis method. J. Optoelectron. Adv. Mater..

[40] Tauc J., Grigorovici R., Vancu A. (1966). Optical properties and electronic structure of amorphous germanium. Phys. Stat. Sol..

[41] Oktik S. (1988). Low Cost, Non-Vacuum Techniques for the Preparation of Thin/Thick Films for Photovoltaic Applications. Prog. Cryst. Growth Ch..

[42] Bao D., Gu H., Kuang A. (1998). Sol-Gel Derived C-Axis Oriented ZnO Thin Films. Thin Solid Films.

[43] Urbach F. (1953). The Long-Wavelength Edge of Photographic Sensitivity and of the Electronic Absorption of Solids. Phys. Rev..

[44] Caglar M., Ilican S., Caglar Y. (2009). Influence of Dopant Concentration on the Optical Properties of ZnO: In Films by Sol-Gel Method. Thin Solid Films.

[45] Swanepoel R. (1983). Determination of the thickness and optical constants of amorphous silicon. J. Phys. E: Sci. Instrum..

[46] Ashour A., Kaid M.A., El-Sayed N.Z., Ibrahim A.A. (2006). Physical properties of ZnO thin films deposited by spray pyrolysis technique. Appl. Surf. Sci..

[47] Maissel L.I., Glang R. (1970). Hand book of Thin Film technology.

[48] Mayadas A.F., Shatzkes M. (1970). Electrical-resistivity model for polycrystalline films: the case of arbitrary reflection at external surfaces. Phys. Rev. B.

[49] Gordon R.G. (1996). Preparation and properties of transparent conductors. Mater. Res. Soc. Symp. Proc..

[50] Huang Y., Liu M., Li Z., Zeng Y., Liu S. (2003). Raman spectroscopy study of ZnO-based ceramic films fabricated by novel sol-gel process. Mater. Sci. Eng. B.

[51] Yahia S.B., Znaidi L., Kanaev A., Petitet J.−P. (2008). Raman study of oriented ZnO thin films deposited by sol-gel method. Spectrochim. Acta A Mol. Biomol. Spectrosc..

[52] Alim K.A., Fonoberov V.A., Balandin A.A. (2005). Origin of the phonon frequency shifts in ZnO quantum dots. Appl. Phys. Lett..

[53] Alim K.A., Fonoberov V.A., Shamsa M., Balandin A.A. (2005). Micro-Raman investigation of optical phonons in ZnO nanocrystals. Appl. Phys. Lett..

[54] Wang L., Pu Y., Chen Y.F., Mo C.L., Fang W.Q., Xiong C.B., Dai J.N., Jiang F.Y. (2005). MOCVD growth of ZnO films on Si(111) substrate using a thin AIN buffer layer. J. Cryst. Growth.

[55] Ismail A.I., Abdullah M.J. (2013). The structural and optical properties of ZnO thin films prepared at different RF sputtering power. J. King Saud. Univ. Sci..

